# Study of Antibacterial Activity of Root Bark, Leaves, and Pericarp Extracts of *Diploknema butyracea* and Evaluation of Prospective Antioxidant Activity

**DOI:** 10.1155/2022/6814901

**Published:** 2022-03-23

**Authors:** Alisha Karki Chhetry, Subhila Dhakal, Lalita Chaudhary, Khimdhoj Karki, Ram Bahadur Khadka, Gautam Prasad Chaudhary, Tonking Bastola, Amrit Poudel, Pramod Aryal, Jitendra Pandey

**Affiliations:** ^1^Department of Pharmacy, Crimson College of Technology, Pokhara University, Devinagar-11, Butwal 32900, Nepal; ^2^Department of Laboratory Sciences, Crimson College of Technology, Pokhara University, Devinagar-11, Butwal 32900, Nepal; ^3^Department of Ophthalmology, University of California, San Diego, La Jolla, CA, USA; ^4^Department of Biodiversity and Bioresources, Satvik Nepal, Dandakonak, Pokhara, Kaski 33700, Nepal

## Abstract

This study was aimed to determine the antibacterial activity of root bark, leaves, and pericarp extract of *Diploknema butyracea* and to evaluate the prospective antioxidant activity, total flavonoid, polyphenol, and carbohydrate content. The plant parts were collected and extracted by cold maceration, using hexane, ethyl acetate, methanol, and distilled water. Phytochemical screening of different samples of *D. butyracea* in different solvents revealed the presence of varied extent of alkaloid, saponin, terpenoid, anthraquinones, tannin, cardiac glycoside, flavonoid, carbohydrate, polyphenol, protein and amino acid, resin, and phytosterol. Our study showed that methanolic root bark extract exhibited the potent antimicrobial activity against *Staphylococcus aureus*, *Staphylococcus epidermidis*, and *Klebsiella pneumonia* with an average zone of inhibition of 17.33 mm, 14.33 mm, and 13.0 mm, respectively. Surprisingly, all of the extracts were insensitive to *Escherichia coli*. The lowest minimum bactericidal concentration (MBC), 4.6 mg/ml, was observed with the aqueous pericarp extract against *S. epidermidis* and the highest was of 50 mg/ml shown by ethyl acetate pericarp against *K. pneumonia*. Our results showed that both the polar and nonpolar components present in the different parts of *D. butyracea* exhibit prominent antibacterial activities against different bacterial strains. The *in vitro* 2,2′-diphenyl-1-picrylhydrazyl (DPPH) free radical scavenging activity showed that the methanol extract of root barks displayed the most potent antioxidant activity (IC_50_ : 6.1 *µ*g/ml). The total polyphenol content of the plant part extracts was observed between 19.48 ± 0.23 and 123.48 ± 1.84 *µ*g gallic acid equivalent/mg of dry extract weight. Likewise, flavonoid content ranged from 40.63 ± 1.28 *µ*g to 889.72 ± 3.40 *μ*g quercetin equivalent/mg of dry extract weight and total carbohydrate content ranged from 11.92 ± 0.60 *µ*g to 174.72 ± 0.60 *µ*g glucose equivalent per/mg dry extract weight. Overall, our study showed that the root bark, pericarp, and leaves extract of *D. butyracea* evinced prominent antibacterial properties against various pathogenic bacterial strains.

## 1. Introduction

Herbal-based traditional medicines have always been a part of human culture since the ancient time [1]. In the modern era, medicinal plants are considered as the center of attention for enormous investigation of their inherent biological effect [2]. Screening of natural plants, targeting specific therapeutic activity, has led to the revelation and discovery of clinically effective medicine to cope with life-threatening human disease [1–4]. In Ayurveda, the use of herbal extracts and nutritional supplements for the treatment of infectious diseases as an alternative or complementary medicine has been well documented and preserved for about 5,000 years. Allopathic medicines can undoubtedly cure a wide range of diseases. However, because of their unavailability, high prices, and unwanted adverse effects, many patients prefer to adopt the natural source of remedy [5]. In the current scenario, bacterial infectious diseases are a serious worldwide public health problem due to irrational use of antibiotics. As a result, diverse classes of multidrug resistant bacterial strains are being generated nowadays [6]. Increased rates of mortality and morbidity are due to the lack of long-term effective drugs and unaffordable cost of new generation antibiotics [7]. The problem of microbial resistance is growing and the prospect of the use of antimicrobial drugs is uncertain. This disastrous situation has compelled us to explore more successful antimicrobial agents using plant resources so that they will serve as an active therapeutic ingredient and lead molecules to the synthesis of optimized new drugs [8].

**Figure 1:**
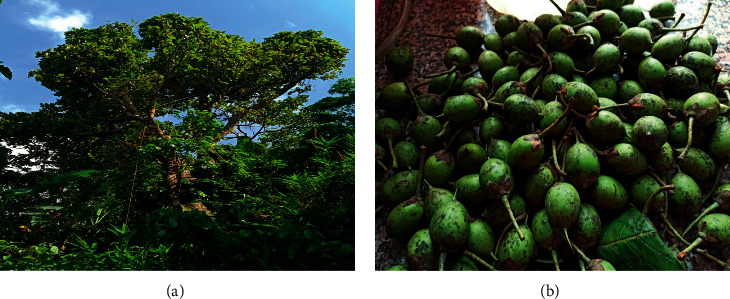
*D. butyracea* plant: (a) whole plant and (b) unripe fruit.

*Diploknema butyracea*, commonly known as the Indian butternut tree in English, is a medium-sized deciduous tree of about 20 m in height (Figure 1) [9, 10]. It belongs to family Sapotaceae and is widely distributed in the tropical and temperate regions, at an altitude of about 300–1500 m, primarily on hill slopes and cliffs [11], and is found in northern India, Tibet, Nepal, Bangladesh, Sri Lanka, and Bhutan [12]. It is popular with the name “Chyuri” in Nepal, “Indian-butter nut” in English, and “Chiura” or “Phulwara” in India [9, 13]. The important ethnomedicinal assets of this plant are seeds, which are utilized for the production of butter or fat, known as Chyuri ghee and it has diverse uses including cooking and lighting lamps by the local communities [14]. Apart from that Chyuri seed butter has also been used to make cosmetic items, soaps, cosmetics, and other commercial products like cooking ghee and candles [9, 11, 15]. The ripen fruits are crushed and applied on topical areas for the treatment of skin ailments in animals as well as human beings.

The bark juice of the plant is widely utilized to cure rheumatism, indigestion, asthma, ulcer, itching, allergy, diabetes, and tonsillitis [9, 11]. The dried powder of stem bark is taken orally by mixing with water or milk to mitigate fever [11, 16]. Dried powder of flower and petals is consumed as a tonic, for the soothing effect of the irritated throat and for increasing lactation. Flowers are an excellent source of honey production. The paste of fresh leaves is used to treat ulceration of the mouth and muscular pain [11, 16, 17]. The prime chemical constituents of Chyuri butter are triglycerides. Major fatty acids found in it are methyl ester form of saturated stearic acid (2.4%), saturated palmitic acid (66%), polyunsaturated oleic acid (2.6%), and monounsaturated linoleic acid (26%) [9]. Besides, different feeding deterrent saponins, MI-III and MI-I [18], and aromatic components such as methyl-2-furoate, heptane, 3,4-dimethyl-1,2- cyclopentadiene, lauryl alcohol, and trans,trans-2,4-heptagonal are also present in the fruit [19]. Diverse pharmacological effects such as the antioxidant effect of fruit pulp [20], antifungal activity of seed extract [21], anti-inflammatory effect [22], and antibacterial activity [23] of stem bark extract along with feeding deterrent and insect growth inhibitory effect of seed extract [18] have been reported for this plant. However, there is no scientific claim on the antimicrobial and antioxidant effects of *D. butyracea* pericarp, root bark, and leaves till date. Thus, this study was aimed to determine the antibacterial activity of root bark, leaves, and pericarp extract of *D. butyracea* and to determine their antioxidant activity, total flavonoid, polyphenol, and carbohydrate content with phytochemical screening.

## 2. Materials and Methods

### 2.1. Drugs and Chemicals

Gentamicin and Ciprofloxacin (Microxpress, a division of Tulip Diagnostics (P), Ltd.) antibiotic discs were used as standard drugs for antimicrobial activity. Mueller Hinton Agar (MHA) (HiMedia Laboratories Pvt. Ltd., Mumbai), Nutrient Broth (HiMedia Laboratories Pvt. Ltd., Mumbai), DPPH (HiMedia Laboratories Pvt. Ltd., Mumbai), Barium Chloride (Thermo Fisher Scientific, India Pvt. Ltd., Mumbai), and Dimethyl Sulfoxide (Thermo Fisher Scientific, India Pvt. Ltd., Mumbai) were also used.

### 2.2. Test Organisms

To investigate the *in vitro* antimicrobial potency of all the plant extract, gram-positive bacteria: *S. aureus* (ATCC 9144) and *S. epidermidis* (ATCC 12228) and gram-negative bacteria: *K. pneumonia* (ATCC 4352) and *E. coli* (ATCC 14948) were collected from S.E.E.D. Laboratory, Rupandehi, Nepal.

### 2.3. Plant Materials

The fresh root barks, leaves, and unripe fruits of *D. butyracea* were collected from Palpa district, Lumbini Province, Western Nepal (1,350 m above the sea level) during August, 2021. The collected plant materials were identified and authenticated from National Herbarium and Plant Laboratory Godawari, Nepal (Ref-078/079). The herbarium of the plant was prepared and preserved in Pharmacognosy Laboratory of the Crimson College of Technology, Butwal-13, Rupandehi, Nepal (Specimen number: CCT/HRB/2021-008).

### 2.4. Plant Extracts Preparation

Firstly, the collected leaves, root barks, and unripe fruits were washed with fresh distilled water. Unripe fruits of *D. butyracea* were first separated into flesh and seeds. Only flesh (pericarp) was chopped into small pieces and left for shade drying for 2 weeks. A similar procedure was adopted for the bark. However, leaves were directly left for shade drying without cutting. The naturally air-dried leaves and root bark were comminuted with a grinder to a fine powder and passed through the #40 mesh sieve (0.381 mm of pore size). Because of the sticky nature of the dried fruit slices, we performed their direct extraction.

In order to ensure the optimal extraction of the plant parts, we used triple cold maceration process. After single maceration with periodic manual shaking in every 6 h for 72 h, the menstruum was collected and marc was further extracted with the same amount of fresh solvents. The whole procedure was repeated three times. Briefly, 200 g each of leaves, root barks, and fruit pericarp of *D. butyracea* were macerated with 1,000 ml of hexane, ethyl acetate, methanol, and water. The liquids from each step of maceration were strained, filtered and pooled and dried at 40°C to obtain a gummy concentrate using rotatory evaporator, and the extracts were stored in refrigerator at 4 ± 1°C until use.

### 2.5. Extractive Yield Value

The extractive yield of *D. butyracea* root bark, leaves, and pericarp in hexane, ethyl acetate, methanol, and water was calculated by using the following equation:

(1) $$\begin{array}{c} \text{Extractive yield}=\frac{\text{Weight of the extract obtained}\left(\text{g}\right)}{\text{Weight of crude drugs used for extraction}\left(\text{g}\right)}\times 100\%. \end{array}$$

### 2.6. Phytochemical Screening

Phytochemical screening of the extracts was performed to identify the presence of various secondary metabolites, namely, alkaloids: Mayer's test; anthraquinone, saponin, flavonoid, resin, and polyphenol: Ferric chloride test; terpenoids and cardiac glycosides: Fehling's test; and phytosterols: Salkowski's test, using standard methods following specific protocols [24–26]. The presence was indicated with + sign whereas absence was indicated with − sign.

### 2.7. Antibacterial Activity Test [23, 27, 28]

#### 2.7.1. Preparation of Plant Extracts and Filter Paper Discs

For each plant extract, 100 mg was taken accurately in a closed small tube and dissolved thoroughly in 1 ml DMSO with the help of sonication. Then, fully dissolved samples were stored in a safe place until use. Each 10 *µ*l of sample solution contained 1 mg of plant extract. Approximately 5 mm diameter of filter paper disc (from Whatman's No. 1 filter paper) was prepared and sterilized for 15 min at 115°C.

#### 2.7.2. Muller Hinton Agar (MHA) Media Preparation and Subculture of Bacterial Strains

The antimicrobial activity was measured by the disc diffusion method. 38 g of MHA was suspended in 1000 ml distilled water in a conical flask. The media dissolved completely and sterilized in an autoclave at 121°C for 15 min at 15 lbs pressure. The hot conical flask media was allowed to cool to 40–50°C in sterilized laminar airflow. The media was poured into each Petri plate and dropped to set. Two hardened media were incubated at 37°C for 24 h to check the possible contamination, and the remaining was refrigerated at 5°C. For subculture, the inoculating loop was inflamed in a burner flame to transfer the bacteria sample to the agar plates. The inoculating loop was cooled and dipped inside the tube to pick up the microorganism. Then, the loop was streaked across the surface of the agar plate in a zigzag pattern. In this manner, all the test organisms were subcultured in separate agar plates with proper labeling. The subcultured plates were incubated at 37°C for 24 h before inoculation. All the experiments were completed in aseptic condition with laminar airflow.

#### 2.7.3. Preparation of Bacterial Suspension/Inoculum

Initially, nutrition broth media was prepared and sterilized. After that, 5 ml nutrient broth was poured into four different sterilized test tubes. Bacterial suspensions of *S. epidermidis*, *S. aureus*, *E. coli*, and *K. pneumonia* were prepared to suspend bacteria (from subculture media) with the inoculating loop to each respective test tube and incubated at 37°C for 24 h. The turbidity of the inoculums suspension was compared with 0.5 McFarland solutions.

#### 2.7.4. Screening and Measurement of Zone of Inhibition (ZOI)

A sterile cotton swab stick was dipped into the turbidity-adjusted bacterial suspension. After that, the dried surface of the media plate was inoculated by rubbing the cotton swab stick (loaded with microorganisms) over the entire sterile media surface. The same technique was repeated for each microorganism. Finally, media plates were divided into four equal parts to insert the standard antibiotic disc and filter disc, containing sample extracts, blank control, in equal distance. 10 *µ*g/disc of Ciprofloxacin and Gentamycin were used for gram-negative gram-positive bacteria, respectively. To load the test sample, 10 *µ*l of each extract (1 mg of extract per disc) were poured into two paper discs (doublet manner) and the third paper disc was used for negative control (10 *µ*l DMSO). All the plates were incubated at 37°C for 24 h. All the measurements were examined in triplicate. After 24 h of incubation, the culture media was taken out from the incubator, and the inhibited areas (ZOI) by the different extract and antibiotics were measured in mm, with the help of digital Vernier Caliper.

#### 2.7.5. Determination of MIC and MBC

The twofold serial broth microdilution technique was adopted to calculate the MIC values of all the plant extract, against four different test organisms. A total of 10 vials were labeled and sterilized; then, 750 *µ*l of sterilized Mueller-Hilton Broth (MHB) was transferred into each vial. For the sample solution preparation, 200 mg/ml of stock solution was prepared in DMSO, subjected to serial dilution, using a 1 : 1 mixture of DMSO and water to prepare sample solutions of 10 different concentrations (200 mg/ml–0.390625 mg/ml). After that 250 *µ*l of sample solution was transferred into a corresponding vial containing 750 *µ*l of MHB, so that the final concentration of sample ranged from 50 mg/ml to 0.09765 mg/ml. Bacteria with an inoculum of about 1 × 10^5^ CFU/ml were loaded into each vial. For the preparation of microorganism inocula, broth culture was incubated for 12 h, and turbidity of the suspension was adjusted to the turbidity of 0.5 McFarland standards. One inoculated vial was used as a negative control, to ensure broth suitability for growth of microorganisms. Also, 4% DMSO was tested as a blank control. After the incubation of the sample containing broth media, for 24 h at 37°C, the MIC value was determined. MIC was taken as the lowest concentration that prevented the visible growth of the bacterial culture. The easy technique to observe the inhibition of growth is the absence of turbidity in the examined tubes. But, it was very challenging to ensure whether the turbidity was due to the nature of plant extract or due to the growth of the bacteria. Thus, MBC was investigated to determine the minimum concentration of the plant extract that can completely kill the tested microorganisms.

For the MBC determination, the refrigerated MHA Petri plates were incubated at 37°C for 45 min and transferred into the sterilized laminar airflow (LAF) hood. After that, samples from each diluted test tubes (obtained after MIC examination) were subcultured on MHA plates followed by incubation for the next 24 h at 37°C. Finally, the minimum concentration of plant extract that completely prohibited the microorganism growth over media surface was noted as the MBC.

### 2.8. Antioxidant Activity Determination

The antioxidant activity of plant extract was checked by using DPPH (1,1-diphenyl-2-picrylhydrazyl) free radical scavenging activity, according to previous methods with slight modification [29–31]. At first, the stock solution of 0.1 mM of DPPH, 1 mg/ml of ascorbic acid, and test solutions were prepared in ethanol. Ascorbic acid solution thus prepared was diluted into different concentrations (10 *µ*g/ml, 5 *µ*g/ml, 2.5 *µ*g/ml, and 1 *µ*g/ml). For the DPPH assay, 4 ml of different extract solutions (31.25 *μ*g/ml, 62.5 *μ*g/ml, 125 *μ*g/ml, 250 *μ*g/ml, 500 *μ*g/ml, and 1000 *μ*g/ml) of the sample was mixed with 4 ml of DPPH solution (0.1 mM) and incubated in dark place. After 30 min, the absorbance of the sample mixture was monitored at 517 nm, with the help of a UV spectrophotometer. Methanol and ascorbic acid were chosen as negative and positive controls, respectively. All the measurements were examined in triplicate. The free radical inhibition percentage was determined calculated using the following formula:

(2) $$\begin{array}{c} \text{Percentage radical scavenged }=\left[\left({\text{A}}_{0}-{\text{A}}_{1}\right)/{\text{A}}_{\text{0}}\right]*100\%, \end{array}$$

where *A*_0_ is the absorbance of DPPH solution and *A*_1_ is the absorbance of the sample.

### 2.9. Determination of Total Phenolic Content, Total Flavonoid Content, and Total Carbohydrate Content

Total phenolic content was determined using the Folin-Ciocalteu (FC) method with a trivial modification of previous research, using gallic acid as a standard. In the study, different concentrations of gallic acid were prepared. The extract solution of 1 mg/ml concentration was made from the ethanolic stock solution. 1 ml of ethanolic stock solution was treated with 1 ml (2N) FC reagent followed by 5 ml distilled water and was shaken for 5 min. Subsequently, 1 ml of 10% Na_2_CO_3_ was added and incubated for 1 h at room temperature. The absorbance was measured utilizing a UV Spectrophotometer at 765 nm against a blank (without extract). All the measurements were evaluated in triplicate [29].

The total flavonoid content was determined using the method used in similar research study. A standard flavonoid compound was quercetin. Different concentrations of quercetin were prepared from the stock solution (1 mg/ml) using ethanol as a solvent. 1 mg/ml concentrations of the pericarp, leaf, and root bark extract were prepared. 1 ml of plant extract was dissolved in 4 ml of distilled water and 0.3 ml of 5% NaNO_2_. After 5 min, 0.3 ml of 10% AlCl_3_ was added and incubated for 5 min. Then, 2 ml of 1M NaOH was added to the solution. Similarly, a blank solution was prepared without a sample. All the reaction mixtures were incubated for 30 min at room temperature, followed by the absorbance measurement at 415 nm, against the blank. All the measurements were examined in triplicate [29].

Total carbohydrate content in different extracts of *D. butyracea* was determined by the phenol-sulphuric acid method, adapted by the previous study. In this test, the standard compound was glucose. Firstly, 1 mg/ml of the stock solution was prepared. The different concentrations of glucose standards (15.625 *μ*g/ml, 31.25 *μ*g/ml, 62.5 *μ*g/ml, 125 *μ*g/ml, 250 *μ*g/ml, and 500 *μ*g/ml) were prepared by serial dilution technique. In 10 ml of the test tube, 2 ml of the sample (1 mg/ml), 1 ml of the 5% phenol solution, and 5 ml of the concentrated sulphuric acid were mixed properly and kept for 10 min. Then, the tube contents were mixed and placed in a water bath at 25–30°C for 20 min. The absorbance readings of the blank and the samples were measured at 490 nm. All the measurements were examined in triplicate [29].

### 2.10. Statistical Analysis

All the experiments were performed three times and the data were presented as mean ± SD. Statistical significance of differences was calculated by one-way ANOVA and Tukey's test.

## 3. Results and Discussion

### 3.1. Extractive Yield Value

The extractive yields of *D. butyracea* root bark, leaves, and pericarp in hexane, ethyl acetate, methanol, and water extract are shown in Table 1.

### 3.2. Phytochemical Screening

A qualitative examination of phytochemical is a key footstep to acquire the scientific information about the presence of medicinally useful secondary metabolites in the plants, revealing a crucial role towards the beneficial medicinal and physiological activities such as antiviral, antimicrobial, anticancer, antioxidant, antidiabetic, and antimicrobial activities [23]. Phytochemical screening of *D. butyracea*, in different solvents, revealed the varied extent of alkaloid, saponin, terpenoid, anthraquinones, tannin, cardiac glycoside, flavonoid, carbohydrate, polyphenol, protein and amino acid, resin, and phytosterol presence. In our study, all the extracts were tannin-free. Protein and amino acid, and anthraquinone were absent in leaf and pericarp extract. Similar results were recorded in other studies [13, 20]. The results are summarized in Table 2.

### 3.3. Antibacterial Test

**Figure 2:**
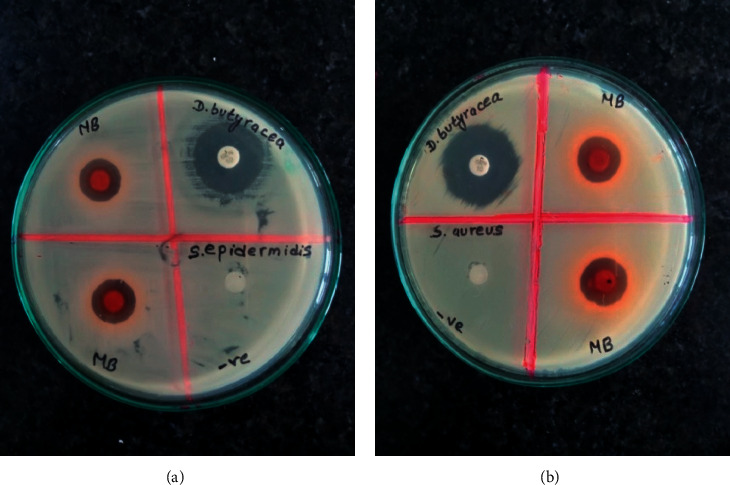
ZOI produced by *D. butyracea* samples: (a) methanolic bark extract against *S. epidermidis* and (b) methanolic bark extract against *S. aureus*.

A total of 12 different extracts, obtained from the leaves, root bark, and pericarp of *D. butyracea*, were screened for their antibacterial activity against four different bacterial strains. Their antibacterial potency was quantitatively confirmed by an inhibition zone absence or presence all over the disc, loaded with the extract. The result confirmed that extracts are more sensitive to gram-positive bacteria in comparison to gram-negative (Table 3). Generally, plant extracts are more active against gram-positive bacteria than gram-negative bacteria due to lipopolysaccharide composition in the multilayered cell wall of gram-negative strains [32, 33]. In this study, methanolic bark extract was reported to be the most significant against *S. aureus* (ZOI-17.33 mm), *S. epidermidis* (14.33 mm), and *K. pneumonia* (13.00 mm). However, the extract remains insensitive against *E. coli*. Also, only pericarp ethyl acetate extract was reported to be sensitive against both gram-negative strains. The ethyl acetate leaves and aqueous leaves extract flaunted antibacterial activity among the leaves, against *K. pneumoniae.* Between the two gram-positive bacteria, *S. aureus* was more sensitive than *S. epidermidis.* In the case of gram-negative bacteria, plant extracts were more effective against *K. pneumonia* than *E. coli*.  Figure 2 depicts the ZOI produced by methanolic bark extract against two gram-positive strains.

**Figure 3:**
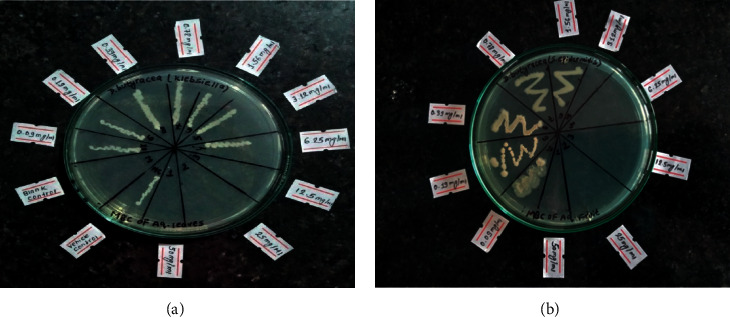
MBC of *D. butyracea* samples: (a) aqueous leaves against *K. pneumoniae* and (b) aqueous pericarp against *S. epidermidis*.

Total 17 samples showed measurable ZOI, which were further screened for MIC and MBC. However, MIC could not be quantified because of the uncertainty of whether turbidity was due to the bacteria growth or due to the plant extract. Thus, MBC was calculated and expressed as mg/ml. The MBC values of different investigated samples were in the range from 4.16 mg/ml to 50 mg/ml. The maximum MBC value of 50 mg/ml was exhibited by ethyl acetate pericarp extract against *K. pneumonia* and the minimum, i.e., 4.16 mg/ml, by aqueous pericarp extract against *S. epidermidis*. The methanolic bark was able to kill both gram-positive strains as well as gram-negative strain *K. pneumonia* at the same concentration, i.e., 25 mg/ml. However, only ethyl acetate extract of pericarp could kill *E. coli* (12.5 mg/ml). All the results are depicted in Table 4. Similarly, Figure 3 shows the MBC shown by two different extract against different bacterial strains. Although extensive studies on bioactive phytochemicals of *D. butyracea* have not been conducted yet, some studies have reported the presence of feeding deterrent saponins 3-O-*β*-D-glucopyranosyl-glucopyranosyl-glucopyranosyl-16-R-hydroxyprotobassic acid-28-O-[ara-xyl-ara]-apiose (MI-III) and 3-O-[*β*-D-glucopyarnosyl-*β*-D-glucopyranosyl]-16-R-hydroxyprotobassic acid-28-O-[ara-glc-xyl]-ara (MI-I) in the *D. butyracea* seed methanolic extract [18]. Also, various antibacterial triterpenoids such as the presence of chemical constituents like triterpenoids (*α*-amyrin acetate, *β*-amyrin acetate, and friedelin) were reported from the bark of *D. butyracea* [34]. These compounds might be responsible for the antibacterial effect. However, bioassay-guided fractionated isolation is necessary to identify the antibacterial compounds present in this plant.

### 3.4. Antioxidant Potency Determination by DPPH Radical Scavenging Activity

**Figure 4:**
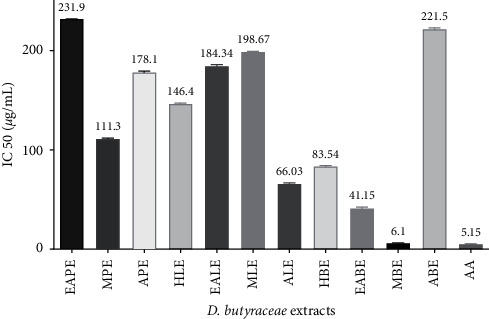
IC_50_ values of different extracts of pericarp, leaves, and root bark of *D. butyracea* extracts along with ascorbic acid. Abbreviations: (HPE: pericarp hexane extract; EAPE: pericarp ethyl acetate extract; MPE: pericarp methanolic extract; APE: pericarp aqueous extract; HLE: leaves hexane extract; EALE: leaves ethyl acetate extract; MLE: leaves methanolic extract; ALE: aqueous leaves extract; HBE; root bark hexane extract; EABE: root bark ethyl acetate extract; MBE: methanolic root bark extract; ABE: aqueous root bark extract).

**Figure 5:**
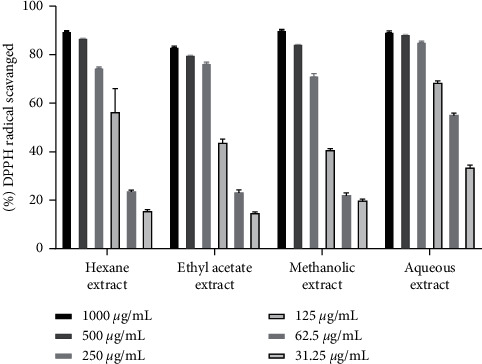
Bar diagram showing DPPH free radical scavenging capacity of *D. butyracea* leaves extract obtained from different solvents at various concentrations.

**Figure 6:**
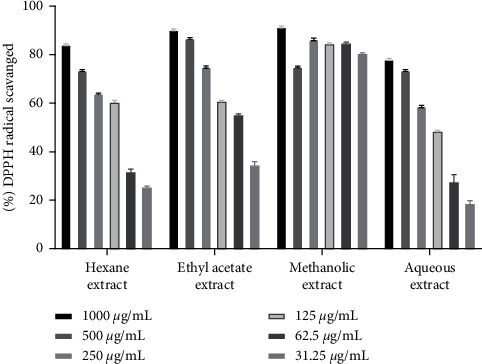
Bar diagram showing DPPH free radical scavenging capacity of *D. butyracea* root bark extract obtained from different solvents at various concentrations.

**Figure 7:**
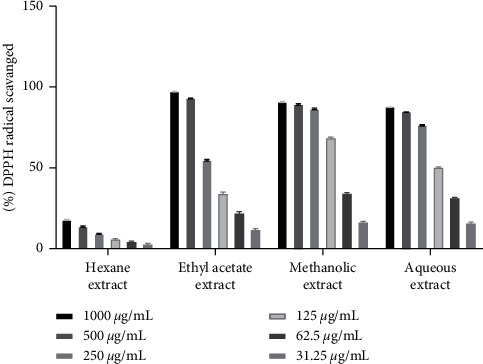
Bar diagram showing DPPH free radical scavenging capacity of *D. butyracea* pericarp extract obtained from different solvents at various concentrations.

The hydrogen atom or electron donation ability of each plant extract against DPPH free radical was measured from the bleaching of violet-colored ethanol solution of DPPH. The DPPH radical absorbs UV radiations at 517 nm. The radical scavenging activity was determined by monitoring the decrease in absorbance [22, 29]. Among three individual parts, our investigation flaunted that *D. butyracea* methanolic root bark extract exhibited the highest capacity to reduce the DPPH free radical (90.52 ± 0.13%) even at the concentration of 200 *µ*g/ml and the lowest scavenging capacity was exhibited by hexane pericarp extract (18.18 ± 0.2% at 1000 *µ*g/ml). Interestingly, the IC_50_ value of methanolic root bark (6.1 *µ*g/ml) was reported to be almost similar to that of standard ascorbic acid (5.15 *µ*g/ml). The IC_50_ value of the *D. butyracea* aqueous stem bark was determined to be 8.43 *µ*g/ml in previous research [22]. In a former study, IC_50_ of the methanolic pericarp (104 *µ*g/ml) [20] was found almost similar to this study (111.3 *µ*g/ml). Among different solvents, the most significant scavenging effect was exhibited by methanolic extract in all the plant parts. On the top, in our study, extract having higher phenolic and flavonoid contents had higher radical scavenging affinity, proportionally. No significant scientific studies have been conducted yet, regarding the antioxidant activity of *D. butyracea* root bark and leaves. The percentage of free radicals scavenged by ascorbic acid at different concentrations is represented in Table 5, whereas Table 6 shows the free radicals scavenged by methanolic bark at diluted concentrations.  Figure 4 represents the IC_50_ values of different samples and standard ascorbic acid. Also, Figures 5–7 depict the bar diagram for free radicals scavenged by *D. butyracea* leaves, root bark, and pericarp, respectively, in different solvents and concentrations.

### 3.5. Determination of Total Phenolic Content, Total Flavonoid, and Total Carbohydrate Content

Polyphenols are abundantly present phytochemical constituents in plants. The hydroxyl group, present in these molecules, can scavenge free radicals. Thus there is a strong correlation between antioxidant potency and the total polyphenol content of many plant species. It has been proven that phenolic compounds are efficient hydrogen donors and serve as a very good antioxidant [35]. In our study, the quantitative estimation of total phenol was accomplished by using Folin-Ciocalteu reagent and the data were expressed as gallic acid equivalent (GAE)/mg of dry extract.  Table 7 shows total phenol content expressed as *µ*g gallic acid equivalent per milligram dry extract weight. There is variation in total phenol content ranging from pericarp hexane extract (18.7 ± 0.23 *μ*g GAE/mg dry extract weight) to methanolic root bark extract (222.16 ± 1.33 *μ*g GAE/mg dry extract weight). From the data of Table 7, it is observed that extraction solvent has a great effect on the phenolic content of the different parts. Also, there is great variation among different plant parts in the same solvent. The statistical analysis showed a significant difference (*p* < 0.05) in the total phenolic content: when each part was compared in different solvents as well as when different parts were compared in the same/each solvent. It is to be noted that the significantly highest phenolic content was recorded in root bark extract whereas the significantly lowest amount was recorded in the hexane extract of the leaf. Also, methanol was found to be the best solvent to extract phenolic compounds significantly in all the investigated parts of the *D. butyracea* plant. Furthermore, the phenolic content of aqueous stem bark determined in a similar study (228.53 *µ*g GAE/mg) [22] was reported to be very high in comparison to the aqueous root bark of our study (62.16 *µ*g GAE/mg). In another study, the total phenolic content of hydromethanolic extract of the pericarp (40.4 *µ*g GAE/mg) [36] was less than methanolic and aqueous (124.6 and 68.97 *µ*g GAE/mg respectively) extract of the pericarp from our study. Phenolic content of leaves extract was reported for the first time.

Flavonoids are a highly diversified and widespread group of natural phenolic compounds. Hydroxyl position present in the flavonoid compounds governs antioxidant properties, and it depends on the electron or hydrogen donation capacity of flavonoid to a free radical [36]. In our study, quantitative determination of total flavonoid was performed by precipitating with aluminum chloride (AlCl_3_) in an alkalinized medium. Results for the total flavonoid content are depicted in Table 7. Among the studied *D. butyracea* samples, there is variation in total flavonoid content ranging from hexane pericarp extract (40.63 ± 1.28 *μ*g QE/mg dry extract weight) to methanolic bark extract 889.72 ± 3.40 *μ*g QE/mg dry extract weight. It is obvious from Table 7 that the extracting solvent has a significant effect on the flavonoid content of the different parts and also each part has different content even in the same solvent. The statistical analysis showed a significant difference (*p* < 0.05) in the total flavonoid content when each part was compared in different solvents as well as when different parts were compared in the same/each solvent. In this study, the order for the flavonoid content in different samples of *D. butyracea* is as follows: root bark > pericarp > leaves. Among the leaves extracts, the highest flavonoid content was found in hexane extract 297.90 ± 0.74 *µ*g QE/mg. Similarly, the highest flavonoid content among the pericarp extract was shown by the ethyl acetate pericarp 649.72 ± 5.60 *µ*g QE/mg. The flavonoid content of all the samples was documented for the first time. Although isolation of flavonoids compounds from the *D. butyracea* leaves, root bark, and the pericarp is not reported yet, quercetin and dihydroquercetin were isolated from the nutshell [37].

Carbohydrates are the abundant organic molecule produced during photosynthetic activity and major structural component of a plant cell. Carbohydrates are the vital energy source that regulates the metabolic processes, stimulates insulin secretion, acts as a powerful neurotransmitter, and alters serotonin concentration [38]. The quantitative determination of total carbohydrate content was carried out using phenol-sulphuric method in terms of glucose equivalent.  Table 7 shows total carbohydrate content expressed as *µ*g glucose equivalent per milligram dry extract weight. There is variation in total carbohydrate content ranging from pericarp hexane extract (11.92 ± 0.60 *µ*g glucose/mg dry extract weight) to methanolic bark extract (174.72 ± 0.60 *µ*g glucose/mg dry extract weight). The result showed that the extracting solvent has a significant effect on the carbohydrates content of the different parts and each part has different content although extracted in the same solvent. The statistical analysis showed a significant difference in the total carbohydrates content when each part was compared in different solvents as well as when different parts were compared in the same/each solvent as mentioned in Table 7. As shown in Table 7, a moderate amount of carbohydrate was detected in the entire sample. Also, carbohydrates got undetected in hexane leaf extract of *D. butyracea*. The methanolic extract of root bark contained significantly the highest amount of carbohydrate among the parts and solvents whereas hexane extract of pericarp has the lowest amount detected.

Notably, our study shows the higher total flavonoid content than the total phenolic content in most of our samples. This observation, however anamulous, is consistent with the similar results from previous studies [39–41]. Our speculation for this anomolous result is that such methods for the specific tests are completely different; the standard used in these two tests is different (we have used quercetin for flavonoid test whereas gallic acid was used for phenolic content test); both methods used for flavonoid and phenol test are not the absolute quantitative measurement, rather they give relative determination in terms of gallic acid and quercetin equivalent, influence of the chemical nature of the flavonoids (such as tannin types of flavonoids) and phenol compounds (such as compounds having less −OH groups on the ring); and total phenolics assay may not detect all the phenolics (as this can depend on the composition phenolic compound) [42, 43]. These might be the possible reasons for higher flavonoid content.

## 4. Conclusion

The present study shows that methanolic extract of *D. butyracea* root bark possesses potent antioxidant and antibacterial activity. It may be due to the polyphenol and flavonoid components. This study highlights that the leaves, bark, pericarp extracts of *D. butyracea* in methanol can be strongly recommended for different biological properties. The study dispenses a prime basis to draw on the extract in the therapeutics of variant maladies. The methanolic extract resonated with the potent antioxidant activity. The root bark, pericarp, and leaves extract of *D. butyracea* revealed evinced prominent antibacterial properties against various pathogenic bacterial strains, recommending the significant utilization in the mitigation of diverse microbial diseases like diarrhea, urinary tract infection, skin infection, dysentery, dental problems, etc. However, further extensive research with great emphasis on the clinical model and the mechanism of action of antibacterial effect is needed to justify ethnomedicinal use of this plant and to pursue the scientific journey of plant-based antimicrobial drug development for safe and effective health care service.

## Figures and Tables

**Table 1:** Percentage yield value of different extracts of *D. butyracea*.

Scientific name	Solvent	Parts used	Sample	Wt. of crude sample (g)	Wt. of dry extract (g)	Yield (%)
*D. butyracea*	Aqueous	Pericarp	APE	46	5.04	10.96
		Leaf	ALE	50	7.16	14.32
		Root bark	ABE	38	9.96	26.22
	Ethyl acetate	Pericarp	EAPE	50	0.57	1.14
		Leaf	EALE	200	6.01	3.00
		Root bark	EABE	50	3.49	6.98
	Hexane	Pericarp	HPE	50	2.75	5.50
		Leaf	HLE	100	4.81	4.81
		Root bark	HBE	50	2.37	4.74
	Methanol	Pericarp	MPE	30	6.52	21.75
		Leaf	MLE	100	10.40	10.40
		Root bark	MBE	119.88	23.91	19.94

APE: aqueous pericarp extract; ALE: aqueous leaf extract; ABE: aqueous root bark extract; EAPE: ethyl acetate pericarp extract; EALE: ethyl acetate leaf extract; EABE: ethyl acetate root bark extract; HPE: hexane pericarp extract; HLE: hexane leaf extract; HBE: hexane root bark extract; MPE: methanolic pericarp extract; MLE: methanolic leaf extract; MBE: methanolic root bark extract.

**Table 2:** Results for the phytochemical screening of root bark, leaves, and pericarp of *D. butyracea* extracted in different solvents.

Leaf					
S. No.	Test	HLE	MLE	EALE	ALE
1	Alkaloid	−	−	−	−
2	Carbohydrate	+	+	−	+++
3	Terpenoid	−	++	−	−
4	Anthraquinone	−	−	−	−
5	Saponin	−	+	+	+++
6	Tannin	−	−	−	−
7	Cardiac glycosides	−	−	−	−
8	Flavonoid	++	+++	−	−
9	Resin	+++	−	−	−
10	Polyphenols	−	++	+++	++
11	Protein and amino acid	++	+++	+++	−
12	Phytosterol	−	+++	−	−
Pericarp					
	Test	HPE	MPE	EAPE	APE
1	Alkaloid	−	+	−	−
2	Carbohydrate	−	−	++	−
3	Terpenoid	−	+	−	−
4	Anthraquinone	−	−	−	−
5	Saponin	−	+	+++	−
6	Tannin	−	−	−	−
7	Cardiac glycosides	−	++	−	−
8	Flavonoid	−	+++	+++	−
9	Resin	−	++	−	−
10	Polyphenols	−	+++	+++	−
11	Protein and amino acid	−	++	++	−
12	Phytosterol	+++	+++	+	+++
Root bark					
	Tests	HBE	MBE	EABE	ABE
1	Alkaloid	−	−	+++	−
2	Carbohydrate	+	+++	+	+
3	Terpenoid	+++	+++	+++	+++
4	Anthraquinone	−	++	−	−
5	Saponin	+	+	+	+++
6	Tannin	−	−	−	−
7	Cardiac glycosides	−	+++	−	−
8	Flavonoid	+	++	+	−
9	Resin	++	−	+	+
10	Polyphenols	++	+++	−	+
11	Protein and amino acid	+	+++	++	−
12	Phytosterol	+	+++	−	−

Abbreviations: *+++:* abundantly present, ++: adequately present, +: less present, −: absent.

**Table 3:** Antibacterial activity of leaves, bark, and fruit extract of *D. butyracea*.

Zone of inhibition in mm (mean ± SD)				
Different samples	*E. coli*	*K. pneumoniae*	*S. epidermidis*	*S. aureus*
HLE	−	−	−	−
EALE	−	8.33 ± 0.57	−	−
MLE	−	−	−	−
ALE	−	10.33 ± 0.57	−	−
HBE	8 ± 1	−	−	8.66 ± 0.57
EABE	−	−	−	−
MBE	−	13 ± 1	14.33 ± 0.57	17.33 ± 0.57
ABE	−	10 ± 0	−	7.66 ± 0.57
HPE	−	−	−	9 ± 0
EAPE	10 ± 1	9.66 ± 0.57	−	11.33 ± 1.15
MPE	−	−	−	10.33 ± 0.577
APE	−	−	8.66 ± 0.57	9.33 ± 0.57
Gentamycin	−	−	20.8 ± 1.30	22.62 ± 2.38
Ciprofloxacin	26 ± 8.5	25 ± 9.5	−	−

*Note.* − indicates inactive in the evaluated concentrations. (Abbreviations: HLE: hexane leaf extract; EALE: ethyl acetate leaf extract; MLE: methanolic leaf extract; ALE: aqueous leaf extract; HBE: hexane root bark extract; EABE: ethyl acetate root bark extract; MBE: methanolic root bark extract; ABE: aqueous root bark extract; HPE: hexane pericarp extract; EAPE: ethyl acetate pericarp extract; MPE: methanolic pericarp extract; APE: aqueous pericarp extract).

**Table 4:** MBC values of leaves bark and fruit extract of *D. butyracea*.

Bacterial strains	MBC values of samples (mg/ml)						
	MBE	ABE	ALE	HPE	EAPE	MPE	APE
*S. aureus*	25 ± 0	−	−	20.83 ± 7.21	25 ± 0	16.66 ± 7.21	20.83 ± 7.21
*S. epidermidis*	25 ± 0	−	−	12.5 ± 0	−	−	4.16 ± 1.80
*K. pneumonia*	25 ± 0	25 ± 0	12.5 ± 0	−	50 ± 0	−	
*E. coli*	−	−		−	12.5 ± 0	−	

*Note.* − indicates inactive in the evaluated concentrations. (Abbreviations: ALE: aqueous leaf extract; MBE: methanolic root bark extract; ABE: aqueous root bark extract; HPE: hexane pericarp extract; EAPE: ethyl acetate pericarp extract; MPE: methanolic pericarp extract; APE: aqueous pericarp extract).

**Table 5:** Percentage inhibition of DPPH free radical by standard (ascorbic acid).

Concentration (*µ*g/ml)	% Scavenged ± SD
1.0 *µ*g/ml	8.87 ± 0.08
2.5 *µ*g/ml	26.98 ± 0.46
5 *µ*g/ml	52.30 ± 0.30
10 *µ*g/ml	92.45 ± 0.18

**Table 6:** Percentage inhibition of DPPH free radicals by methanolic bark extract in diluted concentrations.

Concentration *µ*g/ml	% Scavenged ± SD
0.1 *µ*g/ml	5.90 ± 1.28
1 *µ*g/ml	11.49 ± 0.81
5 *µ*g/ml	41.56 ± 0.26
10 *µ*g/ml	79.79 ± 0.13
100 *µ*g/ml	86.09 ± 0.13
200 *µ*g/ml	90.52 ± 0.13

**Table 7:** Results for the total phenol content of *D. butyracea* leaves, root bark, and pericarp extracted in different solvents.

Different solvent for extraction	Parts	Total phenol content (*μ*g GAE/mg dry extract)	Total flavonoid content (*μ*g QE/mg dry extract)	Carbohydrate content (*µ*g glucose/mg dry extract)
Hexane	Pericarp	79.64 ± 1.01^A^_a_	40.63 ± 1.28^A^_a_	11.92 ± 0.60^A^_a_
Ethyl acetate	Pericarp	113.90 ± 0.26^B^_a_	649.72 ± 5.60^B^_a_	24.14 ± 0.29^B^_a_
Methanol	Pericarp	124.60 ± 0.45^C^_a_	517.30 ± 7.32^C^_a_	105.26 ± 0.60^C^_a_
Aqueous	Pericarp	68.97 ± 0.27^D^_a_	220.93 ± 3.73^D^_a_	88.83 ± 1.19^D^_a_
Hexane	Leaf	18.7 ± 0.23^A^_b_	297.90 ± 0.74^A^_b_	0.00 ± 0.00
Ethyl acetate	Leaf	43.82 ± 1.10^B^_b_	126.39 ± 2.99^B^_b_	43.90 ± 0.88^A^_b_
Methanol	Leaf	194.75 ± 1.57^C^_b_	228.81 ± 0.74^C^_b_	58.33 ± 0.06^B^_b_
Aqueous	Leaf	33.90 ± 0.46^D^_b_	293.06 ± 11.33^A^_b_	49.67 ± 0.56^C^_b_
Hexane	Bark	92.05 ± 0.2^A^_c_	392.45 ± 1.48^A^_c_	23.54 ± 0.32^A^_c_
Ethyl acetate	Bark	94.12 ± 0.44^A^_c_	678.81 ± 4.63^B^_c_	14.03 ± 0.53^B^_c_
Methanol	Bark	222.16 ± 1.33^B^_c_	889.72 ± 3.40^C^_c_	174.72 ± 0.60^C^_c_
Aqueous	Bark	62.16 ± 0.13^D^_c_	287.60 ± 19.83^D^_c_	64.12 ± 0.34^D^_c_

Data were expressed as mean value ± standard deviation (*n* = 3). Different superscripts (A, B, C, and D) within the column represent the significant differences (*p* < 0.05) among the contents of each part (pericarp, leaf, and barks) compared in different solvents (hexane, ethyl acetate, methanol, and aqueous). And different subscript (a, b, and c) within the column represent the significant differences (*p* < 0.05) among the contents of different parts (pericarp, leaf, and barks) compared in each solvent (hexane, ethyl acetate, methanol and aqueous).

## Data Availability

All the data used to support the result of this research are available from Jitendra Pandey and Pramod Aryal upon request.
